# Novel machine learning models to predict endocrine disruption activity for high-throughput chemical screening

**DOI:** 10.3389/ftox.2022.981928

**Published:** 2022-09-20

**Authors:** Sean P. Collins, Tara S. Barton-Maclaren

**Affiliations:** Existing Substances Risk Assessment Bureau, Healthy Environments and Consumer Safety Branch, Health Canada Ottawa, Ottawa, ON, Canada

**Keywords:** estrogen, androgen, (Q)SAR, random forest, *in silico*, machine learning

## Abstract

An area of ongoing concern in toxicology and chemical risk assessment is endocrine disrupting chemicals (EDCs). However, thousands of legacy chemicals lack the toxicity testing required to assess their respective EDC potential, and this is where computational toxicology can play a crucial role. The US (United States) Environmental Protection Agency (EPA) has run two programs, the Collaborative Estrogen Receptor Activity Project (CERAPP) and the Collaborative Modeling Project for Receptor Activity (CoMPARA) which aim to predict estrogen and androgen activity, respectively. The US EPA solicited research groups from around the world to provide endocrine receptor activity Qualitative (or Quantitative) Structure Activity Relationship ([Q]SAR) models and then combined them to create consensus models for different toxicity endpoints. Random Forest (RF) models were developed to cover a broader range of substances with high predictive capabilities using large datasets from CERAPP and CoMPARA for estrogen and androgen activity, respectively. By utilizing simple descriptors from open-source software and large training datasets, RF models were created to expand the domain of applicability for predicting endocrine disrupting activity and help in the screening and prioritization of extensive chemical inventories. In addition, RFs were trained to conservatively predict the activity, meaning models are more likely to make false-positive predictions to minimize the number of False Negatives. This work presents twelve binary and multi-class RF models to predict binding, agonism, and antagonism for estrogen and androgen receptors. The RF models were found to have high predictive capabilities compared to other *in silico* modes, with some models reaching balanced accuracies of 93% while having coverage of 89%. These models are intended to be incorporated into evolving priority-setting workflows and integrated strategies to support the screening and selection of chemicals for further testing and assessment by identifying potential endocrine-disrupting substances.

## Introduction

Endocrine Disrupting Chemicals (EDCs) are a class of chemicals that interfere with human endocrine systems. The toxicity mechanisms for EDCs are diverse; however, certain chemicals may mimic hormones and interact with specific nuclear receptors, such as estrogen receptors (ER) or androgen receptors (AR), leading to endocrine-related effects (Diamanti-Kandarakis et al., 2009; Gore et al., 2015). EDCs are known to cause a wide range of adverse effects, including learning disabilities, cognitive and brain development problems, and cancer (Kajta and Wójtowicz, 2013; Gore et al., 2015). Some notable examples of EDCs are polychlorinated biphenyls, polybrominated biphenyls, and bisphenol A (Diamanti-Kandarakis et al., 2009; Gore et al., 2015). Global regulatory authorities face challenges assessing the hazards and risks they might pose for human and ecological health. The Organization for Economic Co-operation and Development (OECD) has developed guidelines for testing; however, differing legislation, data requirements, and approaches across regulatory frameworks continue to hinder the acceptance and broad integration of novel EDC screening and assessment methods. In response to recommendations by the House of Commons Standing Committee on Environment and Sustainable Development on strengthening CEPA (Canadian Environmental Protection Act) 1999, the Government of Canada is working to improve its ability to screen and consider endocrine disrupting effects when it assesses risks from substances (ECCC, 2018). A key element is the development and consideration of New Approach Methods (NAM) to advance priority setting and risk assessment and to enable a greater focus on substances with an endocrine mode of action. Through deliberations on opportunities to enhance current practices evaluating potential EDCs, the Chemicals Management Plan Science Committee proposed a conceptual workflow that includes a tiered testing and evaluation framework to best deal with the sheer number of substances that need to be considered. Notably, advanced predictive methods, or computational models, are a significant first step (level 1) in the workflow to better address EDCs from priority setting through assessment (Chemicals Management Plan Science Committee, 2018). A tiered framework would include a range of different testing approaches to efficiently guide the prioritization of substances for higher-tier testing and/or risk assessment, as appropriate (Barton-Maclaren et al., 2022). A tiered testing approach can rapidly examine substances triaging those with more significant potential for hazard and/or risk and identify those that merit further investigation at another level where more intensive methods are applied, offering a higher confidence level in predictions about the expected adverse outcome.

*In vitro* high throughput assays, such as accessing the ToxCast Chemicals Inventory database, are an example of level 2 data that can be used or generated in a cost and time-efficient manner compared to *in vivo* testing methods (US Enviromental Protection Agency, 2019). The ToxCast database uses assays, testing a variety of endpoints, and that information can be combined to determine if a chemical is likely to cause endocrine disruption. The assays are typically high throughput and can be conducted on a microliter plate and study a wide variety of toxicological endpoints (US Enviromental Protection Agency, 2021). While the ToxCast database currently has endocrine-related testing results for numerous chemicals, there is a significant portion of chemicals in the Canadian marketplace listed on the domestic substance list (DSL) inventory that have not been tested in the ToxCast program. Furthermore, although the ToxCast assays are considered high throughput, there is still a limit to the number of chemicals that can be tested moving forward, and it is impractical to test all remaining chemicals on the DSL. These testing challenges lead to the need for applying *in silico* models as a first screening step before using high throughput assays for potentially thousands of substances in a prioritization workflow.

A subsection of *in silico* models is machine learning (ML) models, which are computational programs that learn underlying patterns to make predictions. In chemical-based fields, a popular branch of ML models is Qualitative (or Quantitative) Structure-Activity Relationships ([Q]SAR) models) (Q)SAR models, and the broader group ML models, work by using easily calculable values, termed descriptors, to predict activities or properties. For example, in (Q)SAR models used in regulatory toxicology, structural or physical-chemical properties of a substance may be used to predict a toxicological endpoint of concern. In one such example, a variety of (Q)SAR models for different endpoints are available within the OECD (Q)SAR Toolbox (Dimitrov et al., 2016), which uses the chemical structure of substances to make a wide array of predictions, such as blood-brain barrier permeability, dermal absorption, and toxicity flags. There has been focused work in developing computational EDC classification models, including statistically based (Q)SAR models such as ACD (Advanced Chemistry Development) Percepta (Advanced Chemistry Development, 2019), VEGA (Satyanarayan et al., 2016), CaseUltra (Saiakhov et al., 2013), ADMET Predictor (Simulations-Plus, 2020), Oasis TIMES (Todorov et al., 2011), and those presented by Ciallella and co-workers (Ciallella et al., 2020). Some of the most comprehensive work for predicting EDC activity was carried out by the United States Environmental Protection Agency (US EPA), under the Collaborative Estrogen Receptor Activity Prediction Project (CERAPP) (Mansouri et al., 2016) and Collaborative Modeling Project for Androgen Receptor Activity (CoMPARA) (Mansouri et al., 2020).

CERAPP and CoMPARA are models which combine multiple (Q)SAR models to predict ER and AR activity, respectively, and are currently available to use through the freely available OPEn structure-activity Relationship App (OPERA) (Mansouri et al., 2018). The projects include binary and multi-class (based on potency) data for three types of activity; binding, agonism, and antagonism. For CERAPP and CoMPARA, EDC relevant information was gathered from ToxCast and Tox21 (Dix et al., 2007; Richard et al., 2016) in line with approaches for other *in silico* models that used the ToxCast *in vitro* data to make predictive tools (Chushak et al., 2018; Gadaleta et al., 2018; Manganelli et al., 2019; Correia et al., 2021). CERAPP and CoMPARA were developed by providing research groups with training data, using ML methodologies such as k-nearest neighbors, support vector machines, random forests (RFs), and artificial neural networks to develop predictive models. For these methods, a variety of different descriptors were utilized, such as AutoDock scores (Hill and Reilly, 2015), Leadscope (Roberts et al., 2000), DRAGON fingerprints (Todeschini et al., 2007), and GLIDE scores (Friesner et al., 2004). The US EPA weighted and combined the results of those models into a single model known as a consensus model. Consensus models seek to minimize the faults of any one model by combining the predictions from individual models (Papa et al., 2014; Grisoni et al., 2019). Although many models are already developed for EDC prediction, the predictive capabilities of the models on a wide range of various substances are currently unknown. Furthermore, some of these models rely on commercial software, making accessibility challenging if predictions on new substances are required.

In this work, we develop (Q)SAR models to predict endocrine receptor activity, specifically estrogen and androgen receptor activity, to cover a broader range of substances than previous models, aiming to cover more of the Canadian DSL while maintaining high predictive capabilities. Using large datasets from US EPA, RF models were trained and optimized to be incorporated into a priority setting workflow that further compiles, integrates, and interprets various sources and types of information, such as *in vitro* and *in vivo* results as available. Twelve unique models were developed, six for estrogen activity and six for androgen activity, using the Evaluation datasets from CERAPP and CoMPARA, respectively. The six models for each receptor type were binary and multi-class for each activity: binding, agonism, and antagonism. The large datasets provided more data for the models to learn. Descriptors used were from widely available software such as Open Babel and PubChem. These models were developed to be robust, have predictive capabilities over a diverse and considerable number of substances, and be freely available so that any user can readily make new predictions. In addition, models were developed to be conservative, meaning that if the choice is to label a model as Inactive or Active, it will be more likely to be classified as Active. A conservative model will have fewer Active substances classified as Inactive at the expense of more Inactive substances to be classified as Active. The RF models developed in this work will be integrated into a priority-setting workflow to achieve a fuller understanding of the data landscape and toxicological profiles of substance inventories in Canada. These models will be one element of a tiered and integrated framework, providing robust *in silico* information to be weighed with further evidence from *in vitro* and *in vivo* information if available across the chemical space of interest.

## Methods

### Data sources

The information used for model development and testing came from the databases used for the US EPA’s CERAPP and CoMPARA large-scale modeling efforts. The US EPA training data was high throughput screening (HTS) *in vitro* data which was combined using a mathematical model to calculate an area under the curve score, which the authors noted was “roughly proportional to the consensus AC50 value across the active assays” (Judson et al., 2015; Kleinstreuer et al., 2017). In contrast, the original CERAPP Evaluation data, which was used for training the current model, was *in vitro* data collected through literature and available datasets, with the value being an average of the AC_50_ of the collected results for each substance. The choice was made to use the Evaluation dataset to train the RF as the datasets are larger and are expected to cover a broader chemical space, at the expense of the results representing *in vitro* data compared to the training data that aimed to mimic *in vivo*. This was considered a reasonable trade-off as the intended use is within a multi-tiered framework where the RF models will be used to screen a broad and diverse chemical space as an element of an integrated screening and prioritization process. The breakdown of the Training and Evaluation datasets is shown in Tables 1, 2. In this work, the *in vitro* data from the Evaluation datasets were used to develop the models to cover a wide range of chemical space while maintaining high predictive performance. The CERAPP and CoMPARA Training datasets contained 1,812 and 1,855 substances, respectively, with HTS *in vitro* data from the ToxCast and Tox21 programs. The Evaluation datasets had data for 7,522 and 5,064 substances for CERAPP and CoMPARA, respectively, from literature and compiled datasets such as the US Food and Drug Administration Estrogenic Activity Database (Shen et al., 2013) and the ChEMBL database (Gaulton et al., 2012). It should be noted that the data for both the Training and Evaluation datasets for all endpoints were imbalanced, with most substances being labeled Inactive.

**TABLE 1:** Information for binary receptor activity for the training and evaluation datasets.

		Training dataset			Evaluation dataset		
		Binding	Agonist	Antagonist	Binding (≥4 sources)	Agonist	Antagonist
CERAPP	Inactive	1,440	1,458	1,636	5,301 (5,051)	5,969	6,255
	Active	237	219	41	1,982 (350)	350	284
CoMPARA	Inactive	1,464	1,616	1,366	3,298	4,494	3,539
	Active	198	43	159	440	166	343

For the CERAPP evaluation set, the number of substances for which data was derived from four or more sources is shown in brackets.

**TABLE 2:** Information for classification of receptor activity for the training and evaluation datasets.

		Training dataset			Evaluation dataset		
		Binding	Agonist	Antagonist	Binding (≥4 sources)	Agonist	Antagonist
CERAPP	Inactive	1,488	1,505	1,645	5,042 (5042)	5,892	6,221
	Very Weak	133	122	25	685 (43)	19	76
	Weak	45	41	5	894 (215)	179	188
	Moderate	4	3	1	72 (34)	31	10
	Strong	7	6	1	77 (31)	42	10
CoMPARA	Inactive	1,462	1,616	1,371	3,298	4,494	3,539
	Very Weak	107	12	92	141	17	148
	Weak	61	6	55	216	83	176
	Moderate	2	0	2	15	9	6
	Strong	30	25	5	63	55	10

For the CERAPP evaluation set, the number of substances for which data was derived from four or more sources is shown in brackets.

The number of substances presented in this work and thus used to train the RFs may differ from other sources as the datasets were further curated. The substances were converted to the (Q)SAR ready structures and then compared to each other to minimize duplication of substances (additional details in Supplementary Material). For example, 144 structures were removed from CoMPARA binding data as their structures were duplicated within the dataset. This was done to minimize overfitting models to those structures or confusion of the models if the activities differed.

**FIGURE 1:**
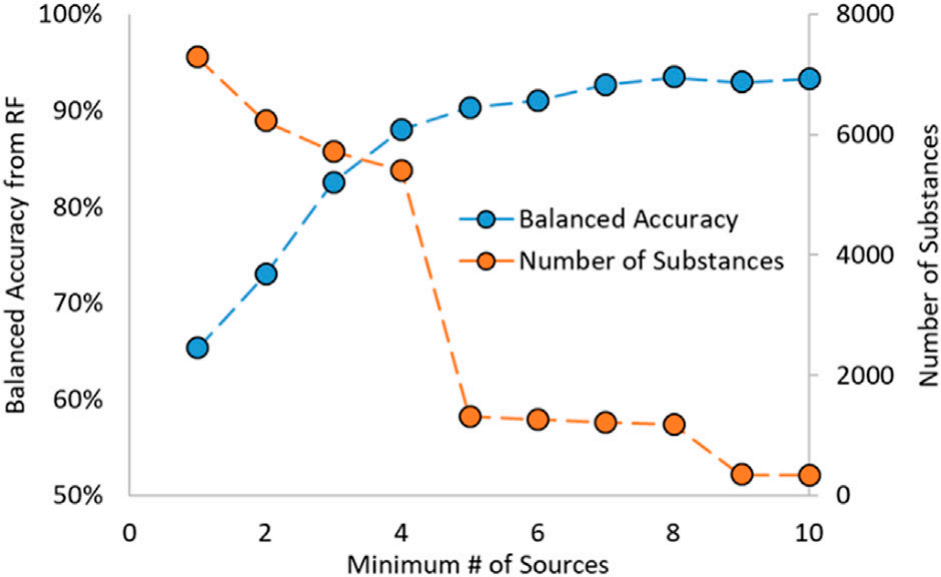
Random Forest Balanced Accuracy and Number of Substances as a function of Minimum Number of Sources.

In work by Mansouri et al., the estrogen activity information was gathered from various sources supported by literature results (Mansouri et al., 2016). Due to the nature of the dataset, most substances have data from multiple sources. Mansouri showed in their work that the predictions made on substances with an increasing number of sources (i.e., a threshold based on the number of sources used) had increased predictive capability. This was also noted during preliminary analysis for the RF model development and shown in Figure 1; as the required number of sources increased, so did the model’s predictive power. Additionally, as the number of minimum sources increased, the number of substances decreased.

A cut-off requiring four or more sources was chosen as it gave the highest balanced accuracy (BA) while also containing many substances. BA is a term used in classification problems, such as for activities of substances, and is the average of the sensitivity (or recall) and specificity. A more generalized term is that the BA is the average of the recalls for each class in a classification problem, shown in the equation below. In that equation, *n* is the number of classes, *i* is the class examined at that time, *TP*_*i*_ is the number of correctly identified substances in class *i*, and *FN*_*i*_ is the number of incorrectly predicted substances in class *i*. The BA is used as it is well-suited for imbalanced datasets (such as the datasets used to train the RF models in this work) and easy and unambiguous to apply to multi-class problems.

$$BA=\frac{1}{n}\underset{i=1}{\sum }\frac{TP_{i}}{TP_{i}+FN_{i}}$$ (1)

### Descriptors

The descriptors were comprised of multiple freely available structural fingerprints and some calculated physical-chemical properties for all chemicals. The structural fingerprints included were PubChem fingerprints (Kim et al., 2019), MACCS fingerprints, and the FP2, FP3, and FP4 fingerprints from OpenBabel (O’Boyle et al., 2011), as well as 15 physical chemistry properties calculated using OpenBabel. Most structural fingerprints are a series of 1’s and 0’s indicating the presence or absence of a chemical feature or substructure, such as if the substance has four or more carbon atoms or contains an aldehyde. The physical-chemical properties included molecular weight, number of atoms and bonds, and predicted values such as melting point and logP. These descriptors were chosen as they cover various substructures and properties. In total, 2,456 descriptors were calculated for each substance and combined to create a single fingerprint. Before the datasets were used to train the RF models, the fingerprints were pruned to remove unnecessary descriptors to make training the models easier and potentially increase the accuracy of RF models. When RF models are trained, each node only tests on a subset of all descriptors to determine the ideal split. By removing low variance and highly correlated descriptors, the remaining descriptors likely contain more relevant information to the models and are represented more often in the descriptor subsets. The first feature reduction step was removing descriptors that had no variance across the datasets. The second pruning step removed descriptors with high collinearity, which was achieved using Pearson correlations. Pearson correlations were calculated between descriptors, and if two descriptors had a Pearson correlation greater than 0.98, only one was used for training.

### Random forest methodology

**FIGURE 2:**
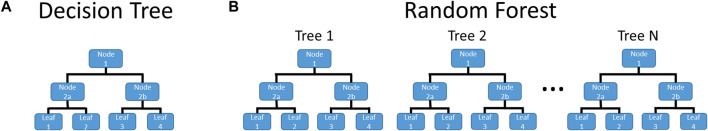
Example of **(A)** a Decision Tree and **(B)** a Random Forest. DTs are a flowchart with each node being a test on an attribute, leading down to a leaf, which gives the prediction. An RF is a collection of DTs, where the final prediction is based on the predictions of the DTs from which it is comprised.

The previously described descriptors and activities were used to train the RF models. The RF code and underlying Decision Trees (DTs) were written in-house using Python programming. A pictorial example of a DT and an RF is given in Figure 2, showing that an RF is a collection of DTs. The RF prediction combines the prediction of its compositional DTs, typically a simple majority voting. The use of in-house codes was done to allow for complete control of the training process. For example, the code allowed the trees to be trained using different cost functions: Gini Impurity, BA, Matthews Correlation Coefficient, and F-Score. For multi-class-based models, BA and F-scores used were macro-averaged, as that helped account for the imbalanced nature of the datasets. In addition, in-house written codes were developed to allow the training protocol to be conservative for screening and prioritization of substances for further exploration. In the case of a tie, conservative predictions were achieved by giving preference to the stronger activity, e.g., equal Inactive and Active predictions would deliver a result of Active, or a tie between Weak and Moderate will generate a result of Moderate. For each RF model, 101 DTs were trained for each combination of the receptor (estrogen or androgen), activity (binding, agonism, and antagonism), the classifier (binary or class), and cost function. At the beginning of training each DT, all non-binary descriptors were binned into twenty equal-sized bins. This is done for each DT, as they are trained on randomly selected subsets of all substances, thereby changing the spacing of each bin. When the non-binary descriptors were tested for splitting the node, the boundary of each bin is tested, with the split being substances below and above the boundary value. For example, if the molar mass range were 50–250, the split would test when substances were above and below 60 g/mol, 70 g/mol, 80 g/mol, and so on up to 240 g/mol. This effectively turned the non-binary descriptors into multiple binary descriptors. Further details of the RF and DT training can be found in the Supplementary Material.

For each RF, after it was trained, an in-house written genetic algorithm (GA) was used to optimize the RF (Whitley, 1994; Mirjalili, 2019). The GA was allowed to alter the depth of each tree independent of all other trees and was even allowed to turn off DTs. By allowing the DTs to be reduced in size or turned off, the RF may be less overfit and give better overall predictions. The GA was designed to improve a scoring similar to the CoMPARA work, which looked at improving the BA of the training and testing data; equations are given in the Supplementary Material (Mansouri et al., 2020). After the RFs were optimized, the single best RF for each combination of receptor, activity, and classifier (binary or multi-class) was chosen as the representative RF for that combination. For example, for binary ER binding, the representative RF was one where the Gini Impurity was used to train the DTs. Forty-eight unique RFs were trained and optimized, with 12 RFs presented in this work.

In addition to optimization of the RF models, applicability domains (ADs) were developed for each model. ADs are a region of chemical space in which accurate predictions about a chemical are likely to be made as the substance is chemically similar to the substances used to train the models. The ADs were calculated using an in-house program that used density k-nearest neighbors (dkNN) neighbors. The AD was developed using only the substances included in each RF model training data. For this approach, the pruned fingerprints were combined using principal components analysis (PCA) (Pearson, 1901), with enough principal components accounting for at least 95% of the fingerprints variance. The methodology and approach of the dkNN were taken from Sahigara et al., who recommend using a threshold cut-off of *n* ^*1/m*^ to consider what is in and out of the AD, where *n* is the number of data points used to create the AD and *m* is a value determined to optimize the AD (Sahigara et al., 2013). To optimize the ADs, the values of *m* were chosen such that the BA would increase, removing a minimal number of substances, typically 15% of the data.

The RF, DT, and AD codes, as well as instructions for installing and running the codes, are available on GitHub under the Massachusetts Institute of Technology Licence (https://github.com/SeanPCollins/RandomForest).

## Results

The RFs and their underlying DTs were designed to give as high predictive power over as wide a range of substances as possible. Another consideration put into the work was to develop models that would give conservative results. This is because the RF models, along with other *in silico* models, would be used as a first tier in a multi-tiered screening and priority setting approach for chemical risk assessment activities. With the RF models designed how they are, (Q)SAR models that cover a broad chemical space were needed to implement into a workflow approach where the results are used in a weight of evidence approach. For substances that require more detailed information (such as *in vitro* or *in vivo*), the (Q)SAR models can be weighted into the overall conclusion. The conservative approach allows for substances that have the potential to be EDC to be flagged to prioritize further information gathering, research, or evaluation. This is seen in the confusion matrix seen in Table 3. The results’ sensitivity and specificity are both high (88.0% and 87.9%, respectively), leading to a BA of 88.0%. However, due to the imbalanced nature of the data sets, there is a high number of false positives (607 out of 915 total positives), leading to low precision of 33.6%. This result may sound poor compared to the sensitivity and specificity; however, this is in line with other models of its type, with precisions ranging from 25.2% to 56.6%. As this method is designed to be a component of an early screening approach, a low false negative rate is more desirable. Confusion matrices for all RF models and performance metrics for all models are given in the Supplementary Material.

**TABLE 3:** Confusion matrix of the estrogen antagonist ER model results when looking at all CERAPP evaluation set substances with four or more data sources.

		Predicted	
		Inactive	Active
Observed	Experimentally Inactive	4,444	607
	Experimentally Active	42	308

In addition to the BA, coverage is an important term that is examined. The coverage is the percentage of dataset substances within the model AD. Higher coverage indicates a greater number, and likely more diverse, substances within the AD. Finally, when applicable, the precision and the recall are also examined. Precision is the ratio of substances predicted active by a model that are observed active. Precision gives an idea of how many substances are falsely predicted active (low precision means a high number of false actives) that, if not high enough, may lead to extraneous work in later stages of the workflow. The recall is the ratio of active substances predicted to be active. The recall is a crucial factor for this work, as these models are intended to be integrated into a priority setting workflow, so it is critical to have a high recall, even at a potential loss of precision.

The results of this work use the CERAPP and CoMPARA Evaluation datasets, which are the same data used to train the RF models. For each RF model training, only 75% of the data was used to train. The remaining 25% was known as the test set for each model and used to validate the models. Therefore, when discussing the performance of the RF models, a focus will be placed on the performance of the models on their respective test sets.

### Binary estrogen receptor activity

**FIGURE 3:**
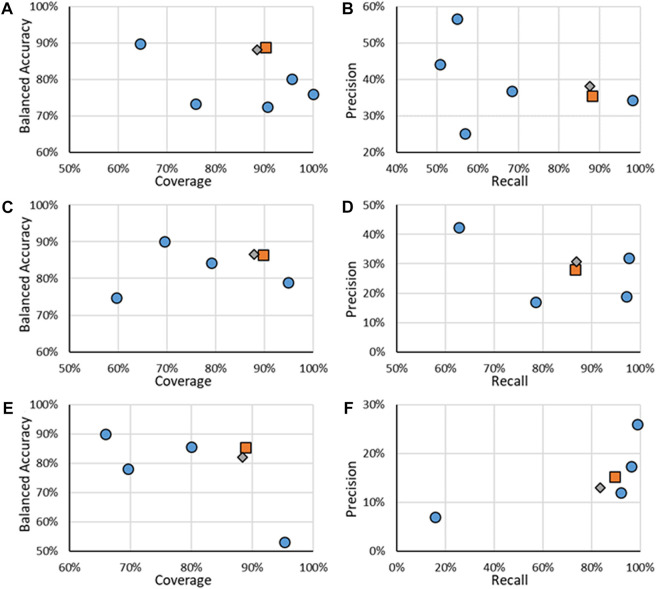
Statistics of performance of the binary ER activity models for binding **(A,B)**, agonism **(C,D)**, and antagonism **(E,F)**. **A, C, and E** show the results of BA as a function of Coverage while **B**, **D**, and **E** show Precision as a function of Recall. Orange squares are for RF models when all substances are considered, gray diamonds are when only the substances in the RF test substances are considered, and blue circles are for other models.

Multiple models, including the novel RF models, were tested using the CERAPP Evaluation data. Statistics of the models are shown in Figure 3, specifically, the BA, the coverage, the precision, and the recall. Specific values for the results are given in the Supplementary Material. The best models would both have a large BA, coverage, precision, and recall, which would be in the upper right corner of each figure. It should be noted that the results for the binding models (Figures 3A,B) showed that only substances which were created from four or more sources were used to evaluate them. The reason to use substances with a minimum number of sources is that it was found that increasing the number of sources leads to an increase in predictive power, which was noted in this work as well as in that by Mansouri et al. (Mansouri et al., 2016). Based on Mansouri’s and our early work, the RF binding models were trained on those high-sourced substances. Results for when all data was included for binding activity are given in the Supplementary Material. For all endpoints, all models were tested on the entire Evaluation dataset from CERAPP (except for removing low-sourced binding information); however, for the RF models, information is also provided for when only substances in their respective test sets were included. For the remainder of the work, statistics of the models discussed will be when their ADs are applied.

For binary ER activity, the RF model results showed good performance, with the lowest BA being 82% and the lowest coverage of 88%. Compared to the other models tested, the RF models appeared to have a good compromise as the results appear closest to the upper right corner of the graph, which is where the best performance is. For ER binding, the RF model is a good compromise between coverage and balanced accuracy and appears to have one of the highest recalls, although at the cost of precision. One model, specifically the CaseUltra ER Binding model, had a higher recall; however, that model had a low coverage of 64.9%. The overall trend appears similar to ER agonism, where the RF model shows a good trade-off between balanced accuracy and coverage and a high recall at the cost of its precision. The two ER agonism models that outperformed the RF model in the recall were the CaseUltra Agonist and Agonist Alpha models; however, their coverages were lower (69.4% and 79.1%, respectively) while having similar or worse precisions. For ER antagonism, the trends were not as consistent. Although the RF model performed well for BA and coverage, it was one of the lowest performing models in terms of recall and precision. The three with better precision-recall performance underperformed in terms of their coverage, with the highest value being 80% compared to the 88.4% of the RF model.

### Multi-class estrogen receptor activity

In addition to the binary models (Inactive/Active), multi-class models were also developed that categorized the potency of the ER effect into five classes from Inactive to Strong. BAs continued to be used as the key statistic for comparison; however, it should be noted that a theoretical minimum BA is the inverse of the number of classes, so for five classes, the minimum is 20%. This is because if all substances were classified as a single activity, that activity would have a 100% recall, but all others would be 0%, for an average of 20%, or the inverse of the number of classes. Confusion matrices for multi-class models are more complex than for binary models due to the increase in classes, with an example shown in Table 4. All multi-class confusion matrices are available in the Supplementary Material. For multi-class models, the BA is the average of each class’s correctly identified (recall or sensitivity) substances, so for the example in Table 4, it would be 72.2%. For tiered multi-class models, such as the predicting strength of an ED activity, some misclassifications are not as problematic as others are. For example, if a Moderate substance is classified as Strong, it is not as concerning as a Moderate active substance being classified as Inactive. In a workflow approach, Inactive substances may be removed from consideration, and both Moderate and Strong substances may move on to the next step in a tiered screening and risk assessment approach. To the best of our knowledge, there does not appear to be a method or statistic to deal with those sorts of errors in the predictions, so analyzing the results will be done using the currently known and used methods where all errors are treated equally.

**TABLE 4:** Confusion matrix for the RF model predicting the multi-class binding potency of the CERAPP Evaluation Dataset.

		Predicted					
		Inactive	Very weak	Weak	Moderate	Strong	Recall (%)
Observed	Inactive	3,416	854	73	128	113	74.5
	Very Weak	3	35	0	2	0	87.5
	Weak	23	72	67	22	5	35.4
	Moderate	0	1	0	24	5	80.0
	Strong	0	0	0	5	25	83.3

**FIGURE 4:**
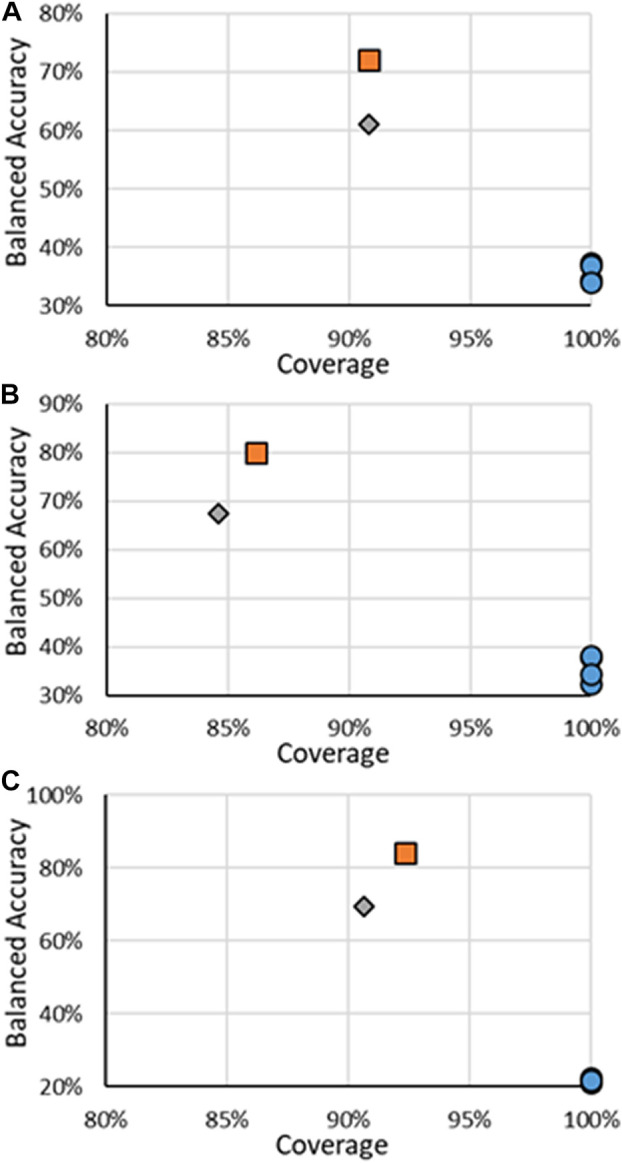
BA vs. coverage for ER multi-class models tested in this work for **(A)** binding, **(B)** agonism, and **(C)** antagonism. Orange squares are for RF models when all substances are considered, gray diamonds are when only the substances in the RF test substances are considered, and blue circles are for other models.

The results for all ER multi-class models are in Figure 4, with specific values given in Supplementary Material. It should be noted that for multi-class models, the definitions of precision and recall are not as well defined as for binary models. For multi-class models, only the BA and coverage will be examined. There were fewer models found for predicting multi-class activity, the RF models developed here and the CERAPP consensus models, so for multi-class models, the individual models that make the CERAPP consensus models were included. The multi-class models were not available in OPERA; therefore, the results for the multi-class models were taken from the original CERAPP work. This means in terms of coverage, the CERAPP and consensus models all had 100%, while the coverage for the RF models ranges from 84.6% to 90.8%. The loss of decrease was seen with a substantial increase in BA values, with the RF models outperforming the best CERAPP models, having balanced accuracies of 23.7–47.0 points (e.g., 22.5% vs. 69.5%) higher. This difference was most noticeable in the antagonism activity. The high BAs of the RF models can likely be attributed to the use of the CERAPP Evaluation dataset to train the models as opposed to the Training dataset that the component models were trained on. This increase in data can be significant for imbalanced multi-class classification problems, such as ER activities. For example, in the CERAPP Training dataset, there was only one Moderate and one Strong antagonist compared to ten and ten, respectively, in the CERAPP Evaluation Dataset. This is a consistent trend for all Active classes across the three activities. The lack of data in each class can make it difficult for any model to learn the underlying patterns of what makes a substance have a particular activity. A marked decrease in the BAs was observed between the RF on all substances and when only testing the Test set substances. This decrease may be due to the overfitting of the models due to a small number of substances, or it could be that a single misclassification of the Test set data can potentially lead to substantial changes in the BA. For example, there are 31 Strong binding substances (when the AD is ignored), and only five of the substances are in the Test Set. Each class contributes 20% to the BA, so if one of the five Strong substances is misclassified, that will decrease the BA by 4%. Another difficulty in multi-class-based models is that not every misclassification has the same meaning when looking at potential outcomes. What is meant by this is if a Strong substance were classified as Moderate, they might still have the same next step applied, such as moving ahead for risk assessment. The same may not be valid for a Strong substance being misclassified as Inactive, where it may not be considered for risk assessment. In this respect, classifying a Strong substance as Moderate could be viewed as a minor error instead of misclassifying a Strong substance as Inactive. To the best of our knowledge, there is not a methodology widely used to account for these errors; therefore, we choose to use the standard BA we used for the binary models.

### Binary androgen receptor activity

**FIGURE 5:**
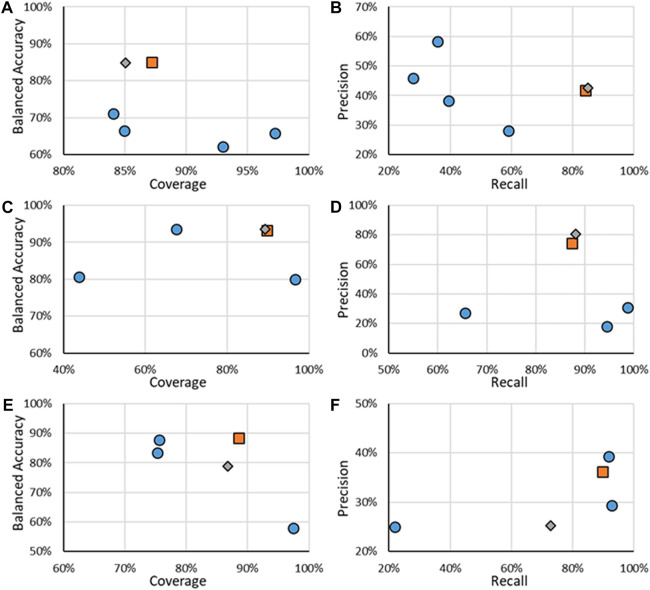
Statistics of performance of the binary ER activity models for binding **(A,B)**, agonism **(C,D)**, and antagonism **(E,F)**. **A, C, and E** show the results of BA as a function of Coverage while **B, D, and E** show Precision as a function of Recall. Orange squares are for RF models when all substances are considered, gray diamonds are when only the substances in the RF test substances are considered, and blue circles are for other models.

In addition to the ER activity, RF models were developed for AR activity; the results for the binary models are shown in Figure 5, with values given in the Supplementary Material. When looking at the BA and coverages (Figures 5A,C,E), the RF models show that coverages do not go below 85%; however, they have ranges of BAs from 78.9% to 93.6%. Some models have higher BAs than the RF models, such as the CaseUltra Antagonist HEK model being 8.8 points higher than the respective RF model; however, this increase is seen with a decrease in the coverage (86.7% compared to 75.6%). A similar trend is also seen in the other direction, such as AR binding where the CoMPARA consensus model had much higher coverage (97.2% compared to 85%), although its BA was much lower, 65.8% compared to 84.9%. There was also a notable difference in performance for the antagonist RF model when comparing the Evaluation and Test sets, the BA dropped from 88.3% to 78.9% and the coverage dropped from 88.5% to 86.7%. This may be attributed to the overfitting of the RF model to the data that even the GA optimization was unable to overcome. Across all AR activities, the RF models appeared to have good trade-offs between coverage and BA.

The precision and recall were also assessed for all binary AR models, with those results shown in Figures 5B,D,F. The RF models had reliable performances for AR binding and agonism predictions with recalls of 85% and 88%, respectively, and respective precisions of 42.5% and 80.4%. These results compared well with the other models having precisions and recalls, which showed good performance. In the example of AR binding, VEGA had the highest precision of 59.1% but with a recall of 28.0%. In comparison, the RF model precision was 42.5% but had a recall of 84.9%. AR agonism shows a similar trend where the CaseUltra Agonist MDA model had a recall of 98.7% compared to the RF recall of 88.1% however its precision was 30.9%, compared to the 80.4% precision of the RF model. AR antagonism showed poor performance compared to other models for recall and precision, where the CaseUltra Antagonist HEK model had a higher recall (91.7% and 72.7%) and precision (39.3% and 30.6%). The RF models perform well when considering all four metrics; BA, coverage, precision, and recall. This is because the RF models are superior at balancing all four statistics while not excelling in any single aspect (apart from precision for the agonist models). The one exception is the performance of the antagonist models, where both versions of the CaseUltra appear to do better in terms of BA, precision, and recall. The only metric in which the RF model outperforms them is the coverage. Overall, the RF models have good statistics demonstrating suitability for use in a workflow to support the identification of priority substances for further evaluation.

### Multi-class androgen receptor activity

**FIGURE 6:**
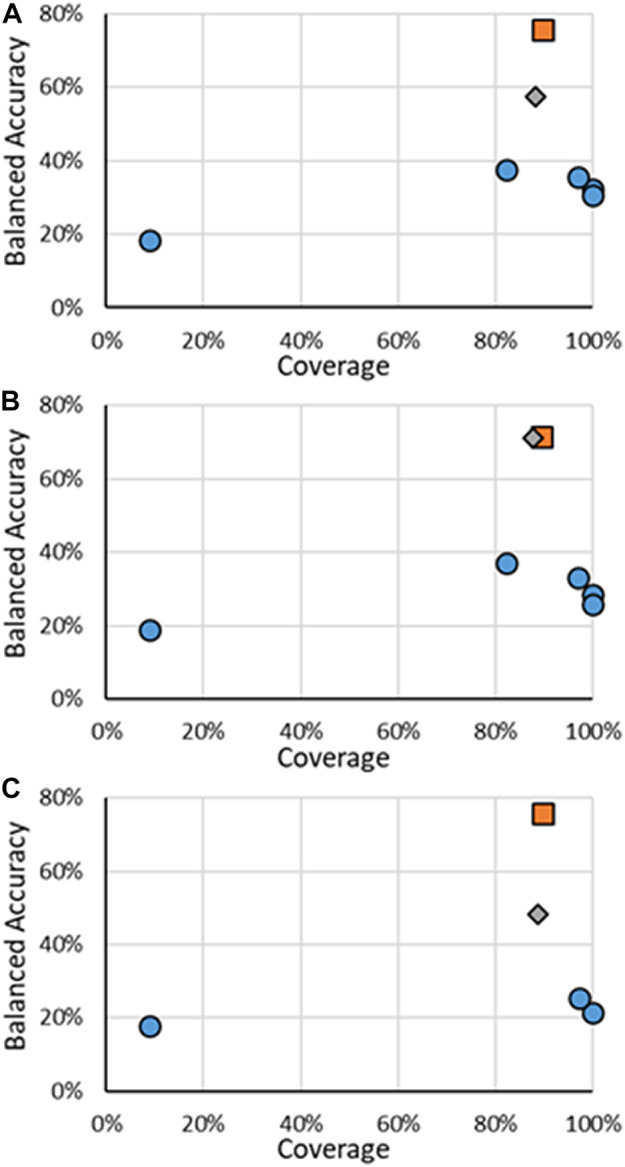
BA vs. coverage for AR multi-class models tested in this work for **(A)** binding, **(B)** agonism, and **(C)** antagonism. Orange squares are for RF models when all substances are considered, gray diamonds are when only the substances in the RF test substances are considered, and blue circles are for other models.

The results for the multi-class models for AR activity are shown in Figure 6, with specific results in the Supplementary Material. It should be noted that, unlike the other activity endpoints, no other published models were found, even from CoMPARA. The US EPA did not develop multi-class models for CoMPARA; however, the results from the submitted models were used for comparison. The multi-class AR activity RF models were the highest performing models developed when considering both the coverage and the BA. The RF models far outperformed the competing models, with the BAs being 19.9% and 34.2% higher than the following closest models. The substantial increase of predictive power of the RF models is seen without much loss of AD size, where ADs for the RF test sets ranged were around 88%, compared to nearly 100% of some of the other models. The overall performance increase can be attributed to the increased size of the dataset used to train the models. For example, there are 43 active agonists in the CoMPARA Training dataset, with 25 labeled as Strong and no Moderate substances, compared to 164 agonists in the Evaluation dataset, 55 labeled as Strong and 9 Moderate. The lack of active agonists in the Training dataset and their bias toward Strong substances meant little information for the CoMPARA models to learn about the other classes.

## Discussion

Using (Q)SAR models can provide valuable information for screening and prioritizing substances for chemical testing and risk assessment. In the context of data-poor substances, incorporating *in silico* and computational approaches can provide predictions about a toxicity endpoint of potential concern for human health on many substances that may otherwise go undetected (Q)SAR models are routinely used in regulatory toxicology (Madden et al., 2020; Cronin et al., 2022), such as the US EPA developing CERAPP for ER activity (Mansouri et al., 2016), CoMPARA for AR activity (Mansouri et al., 2020), or the Danish EPA developing (Q)SAR models for thyroid receptor activity (Rosenberg et al., 2017). These models can rapidly make predictions on models with minimal trade-offs of accuracy. A challenge related to the application of *in silico* models in regulatory contexts is determining those with high predictive capabilities and cover a wide range of chemical space.

The datasets used in this study were large, containing *in vitro* data developed initially by the US EPA for use under CERAPP and CoMPARA and further used to evaluate other (Q)SAR models. The datasets were subject to a consistency check where duplicated information was removed. Additionally the CERAPP dataset binding information had a threshold applied where only substances that contained more than three sources of information were considered, based on the current analysis and findings in the original CERAPP work (Mansouri et al., 2016).

RF models were developed using the US EPA CERAPP and CoMPARA evaluation datasets using in-house developed DT and RF models. After training the RFs, they were further optimized using an in-house developed genetic algorithm, which aimed to maximize the predictive capability of the models, both for the substances they were trained on and an unseen test set of data. After optimization, ADs were developed, and the thresholds were selected to maximize the predictive capability and the number of substances within the AD. This procedure was done for binary and multi-class information for ER and AR binding, agonism, and antagonism, for 12 RF models developed in this work.

The performance of RF models was compared against other *in silico* models across several endocrine disrupting toxicological endpoints, specifically for estrogen and androgen receptors. The number of comparison models varied based on the endpoint. It was found that the RF models were consistently high performing, if not the best performing, models in terms of both BA and coverage. This was most notable in the multi-class models as there were few models for comparison, making them one of the few available. In addition, the models had higher overages and BAs without sacrificing precision or recall too much. The RF models did not have the highest values in terms of precision or recall. However, considering the four statistics in this work, the RF is among the highest performing, if not the highest performing models.

The overall development of this work demonstrates that these models are well suited to fit into a weight of evidence workflow for priority setting of substances for chemical risk assessment activities. These models can provide predictions on substances that may lack higher-level data (such as *in vitro* or *in vivo* studies) while still providing confidence that the substances most likely to have the potential for hazard and risk are detected. Although previous models have been developed for these endpoints, the RF models developed and described here demonstrate high performance compared to a variety of other models tested on the same dataset with a broader AD, providing added value for rapid screening of large chemical inventories.

## Conclusion

The RF models were developed and optimized to give predictions of estrogenicity and androgenicity for substances, given only their structure, offering significant value where there is a need for high throughput screening to gain a better understanding of the potential for toxicity of data-poor substances in the context of prioritization and tiered testing frameworks. The RF model used simple, freely available descriptors, such as PubChem descriptors, and used data from the US EPA CERAPP and CoMPARA projects. For estrogenicity and androgenicity, binary (Inactive/Active) and multi-class (Inactive to Strong) models were developed. A comparative analysis of the models showed that for the studied toxicological endpoints, the RF models developed in this work were the highest performing models when considering the BAs and the coverage. These results were also found without the RF models having a decrease in precision or recall, which is advantageous for models which would be incorporated into an integrated workflow for priority setting. The high performance was especially notable in the multi-class models, which can be attributed to using a large dataset with enough substances to have a reasonable number and distribution across most classes. Overall, the RF models developed and examined in this work provide high accuracy predictions from simple descriptors while covering a diverse range of substances. These models are well suited to support large-scale screening efforts of diverse chemistries to determine if they are likely to be endocrine active and may warrant further exploration for potential as EDCs. These models are good candidates for integrating into a workflow for priority setting and tiered testing and assessment approaches. By implementing the predictions into a workflow that uses a weight of evidence approach, the predictions from these models can be used to support the identification of substances or groups of substances in a priority setting when *in vivo* or *in vitro* is unavailable. Further, the application of in silico and computational approaches offers important evidence for identifying critical data needs in support of future research and information generation efforts to link early indicators of toxicity to adverse outcomes. Future work will investigate finding trends in these models for poor predictions and training the RF model on different endpoints relevant for human health risk assessment, such as acute toxicity and genotoxicity.

## Data Availability

The raw data supporting the conclusions of this article will be made available by the authors, without undue reservation.
