# Supplementary material for: Novel machine learning models to predict endocrine disruption activity for high-throughput chemical screening
Source: Front Toxicol. 2022 Sep 20;4:981928. doi: 10.3389/ftox.2022.981928 (PMC9530987; doi:10.3389/ftox.2022.981928)
Supplement: Supplementary file 1 [file DataSheet1.docx]

Supplementary Information For:

Novel Machine Learning Models for Predictions of Endocrine Disrupting Chemicals for High-Throughput Screening

Sean P. Collins and Tara S. Barton-Maclaren

Existing Substances Risk Assessment Bureau, Healthy Environments and Consumer Safety Branch, Health Canada, Ottawa, ON, K1A 0K9

Table of Contents

[1) Database Curations 3](#_Toc83716486)

[2) Choices to Remove Low Source Estrogenic Binding 3](#_Toc83716487)

[3) Model Development 4](#_Toc83716488)

[4) Confusion Matrices of Estrogen Prediction 5](#_Toc83716489)

[5) Confusion Matrices of Androgen Prediction 8](#_Toc83716490)

[6) References 11](#_Toc83716491)

# Database Curations

The initial step after getting the databases was to curate the databases for similar substances. This was first done in a multiple step process.

1. The SMILES, Names, and CAS-RN provided in the datasets across numerous online databases and gather structural information
   1. CompTox^1^, Pubchem^2^, ChemSpider^3^, US EPA AcToR,^4^ CACTUS, CAS Common Chemistry were all searched
2. The key structural format used throughout the whole process was the International Chemical Information Key (InChI Key)
3. For each substances choose the best (most common) option for structure. This has the possibility of multiple structures, which then the best structure was selected by hand.
   1. Most structures with discrepancies were between different stereochemistry, which is not used in the random forest models
4. After a structure was chosen for each chemical entry, the first 14 characters were used as the structural identifier which contains the connectivity information of the structure.
5. The shortened InChI Keys were then compared among the databases to find similar substances and combine the information when possible.
   1. For some substances, this led to an overall reduction of the total substances within the CoMPARA dataset
6. For the CERAPP dataset, based on information from the work of ^5^ as well as work shown later in this paper, concerning the estrogen binding activity substances with 3 or fewer data sources were removed. When looking at results, this primarily removed FP predictions from the end results of the (Q)SAR models.

# Choices to Remove Low Source Estrogenic Binding

Below

**Table S1.** Binding accuracies for estrogen receptor binding activity for all tested models when all substances and when only those with 4 or more literature sources were evaluated

| Model | Binding (All substances) | Binding (≥4 sources) |
| --- | --- | --- |
| RF Model (Evaluation Set) | 65.3 [65.3] | 88.0 [88.8] |
| RF Model (Test Set) | 87.7 [88.1] | 87.7 [88.1] |
| Best CERAPP Model | 60.8 [65.9] | 81.1 [82.8] |
| CERAPP Consensus Model | 60.7 | 79.9 |
| CaseUltra | [84.3] | [89.8] |
| ACD Percepta | 58.1 | 76.0 |
| ADMET Predictor | [56.6] | [72.6] |
| VEGA | [55.2] | [73.3] |

#

# Model Development

Random Forest (RF) models and the underlying Decision Trees (DTs) were developed in-house to allow greater control and understanding of how the codes worked.

The RF models, and more so the underlying DTs, were trained using the CERAPP and CoMPARA datasets. For each model, before any training occurred, the dataset was pruned to contain only substances which had data available for that activity. After that the descriptors were pruned to remove ineffective or redundant values. This was done by first removing any descriptor which had zero variance across the entire dataset. After the pruning of null variance variable, next all variables were compared to check for co-variance. This was done by calculating the Pearson correlation coefficient and if two descriptors had a correlation coefficient above 0.98 one of the descriptors was removed. The process of pruning could reduce the number of features from 2456 to anywhere between 1264 and 1318 depending on the end point.

An aspect about RFs is the same data, and therefore descriptors, only a subset of descriptors are considered at each node, with the descriptors chosen being randomly selected. The fraction of descriptors chosen was chosen was determined with the equation of 1 – 0.02^1/Number of DTs^, and with the case of 101 DTs, this makes a value of 0.038. The reason this equation was chosen was to give each descriptor a 98% probability to be considered at the 1^st^ node across all decision trees. This was done as the first node has the largest impact on the performance a DT and we wanted to try and give that opportunity to as many distinct descriptors as possible. For the continuous descriptors, such molar mass or logP, at the start of training each DT the continuous variables are divided into 20 bins of equal width. When the descriptor is chosen to test a split on, the DT is allowed to choose to split on any of those bins.

When training the models, the RF model was trained on 75% of the dataset, and 25% was removed to use for testing. The 75% of the dataset was randomly selected. The 75% of the RF training dataset was then used to train the DT models. For each DT model a random 75% of the training data (which itself is 75% of the entire dataset) was chosen to train the DT, while the remaining data was reserved to test the DT. When training the DTs, the model would not try to split a node any further if the node: 1) contained only one class, 2) contained 5 or fewer substances, or 3) did not increase the score by 0.5%. These choices were implemented to prevent over training of the model. Models were trained to optimize the DT Gini and Balanced Accuracy. No cross-validation was performed to train the DT models or the RF models.

After a model was trained, it was then optimized using an in-house developed genetic algorithm (GA). The GA works by altering the depth of each tree, with the potential to turn off a tree. The depths would be set as they were trying to optimize the predictive values as laid out in the works of Mansouri et al.^6^ and shown below. For the multi-class models, for each class the sensitivity and specificity and the median difference between the two were selected as representative for the model, consistent with that of Mansouri et al.^6^ For this work, the balanced accuracy is the average of all SNs for each class as represented by the following equations.

$$S=0.3\left( Goodness of fit \right)+0.45\left( Predicitity \right)+0.25(Robustness)$$

$$Goodness of fit=0.7BA_{Tr}+0.3*(1-\left| Sn_{Tr}-Sp_{Tr} \right|)$$

$$Predictivity=0.7BA_{Eval}+0.3*\left( 1-\left| Sn_{Eval}-Sp_{Eval} \right| \right)$$

$$Robustness=1-|BA_{Tr}-BA_{Eval}|$$

$SN_{i}=\frac{TP_{i}}{TP_{i}+FN_{i}}$ $SP_{i}=\frac{TN_{i}}{TN_{i}+FP_{i}}$ $BA_{i}=\frac{1}{n_{i}}\sum_{j} {SN}_{ij}$

# Confusion Matrices of Estrogen Prediction

Below are the confusion matrices for the binary activity of the RF models developed.

**Table S2.** Confusion matrix for estrogen receptor binding activity from the values seen in the CERAPP evaluation set and the random forest model. Information for all substances with 4 or more sources. Numbers in brackets are when the AD is applied.

|  |  | Predicted | |  |
| --- | --- | --- | --- | --- |
| Observed |  | Inactive | Active | Recall (%) |
|  | Inactive | 4444 (4079) | 607 (489) | 88.0 (89.3) |
|  | Active | 42 (36) | 308 (270) | 88.0 (88.2) |
|  | Precision (%) | 99.1 (99.1) | 33.7 (35.6) |  |

Binding Accuracy: 88.0% or 89.3% when AD is applied.

**Table S3.** Confusion matrix for estrogen receptor binding activity from the values seen in the CERAPP evaluation set and the random forest model. Information for testing set substances with 4 or more sources. Numbers in brackets are when the AD is applied.

|  |  | Predicted | |  |
| --- | --- | --- | --- | --- |
| Observed |  | Inactive | Active | Recall (%) |
|  | Inactive | 1097 (980 | 155 (126) | 87.6 (88.6) |
|  | Active | 12 (126) | 87 (78) | 87.9 (88.1) |
|  | Precision (%) | 98.9 (98.9) | 36.0 (38.2) |  |

Binding Accuracy: 87.7% or 88.1% when AD is applied.

**Table S4.** Confusion matrix for estrogen receptor agonist activity from the values seen in the CERAPP evaluation set and the random forest model. Information for all substances. Numbers in brackets are when the AD is applied.

|  |  | Predicted | |  |
| --- | --- | --- | --- | --- |
| Observed |  | Inactive | Active | Recall (%) |
|  | Inactive | 5116 (4606) | 852 (733) | 85.7 (86.3) |
|  | Active | 50 (44) | 300 (285) | 85.7 (86.6) |
|  | Precision (%) | 99.0 (99.1) | 26.0 (28.0) |  |

Binding Accuracy: 85.7% or 86.4% when AD is applied.

**Table S5.** Confusion matrix for estrogen receptor agonist activity from the values seen in the CERAPP evaluation set and the random forest model. Information for testing set substances. Numbers in brackets are when the AD is applied.

|  |  | Predicted | |  |
| --- | --- | --- | --- | --- |
| Observed |  | Inactive | Active | Recall (%) |
|  | Inactive | 1278 (1115) | 204 (181) | 86.2 (86.0) |
|  | Active | 14 (12) | 83 (80) | 85.6 (87.0) |
|  | Precision (%) | 98.9 (98.9) | 28.9 (30.7) |  |

Binding Accuracy: 85.9% or 86.5% when AD is applied.

**Table S6.** Confusion matrix for estrogen receptor antagonist activity from the values seen in the CERAPP evaluation set and the random forest model. Information for all substances. Numbers in brackets are when the AD is applied.

|  |  | Predicted | |  |
| --- | --- | --- | --- | --- |
| Observed |  | Inactive | Active | Recall (%) |
|  | Inactive | 4968 (4541) | 1286 (1058) | 79.4 (81.1) |
|  | Active | 27 (22) | 257 (190) | 90.5 (89.6) |
|  | Precision (%) | 99.5 (99.5) | 16.7 (15.2) |  |

Binding Accuracy: 85.0% or 85.4% when AD is applied

**Table S7.** Confusion matrix for estrogen receptor antagonist activity from the values seen in the CERAPP evaluation set and the random forest model. Information for testing set substances. Numbers in brackets are when the AD is applied.

|  |  | Predicted | |  |
| --- | --- | --- | --- | --- |
| Observed |  | Inactive | Active | Recall (%) |
|  | Inactive | 1229 (1121) | 337 (275) | 78.5 (80.3) |
|  | Active | 10 (8) | 59 (41) | 85.5 (83.7) |
|  | Precision (%) | 99.2 (99.3) | 14.9 (13.0) |  |

Binding Accuracy: 82.0% or 82.0% when AD is applied.

**Table S8.** Confusion matrix for estrogen receptor binding activity from the values seen in the CERAPP evaluation set and the random forest model. Information for all substances with 4 or more sources. Numbers in brackets are when the AD is applied.

|  |  | Predicted | | | | |  |
| --- | --- | --- | --- | --- | --- | --- | --- |
| Observed |  | Inactive | Very Weak | Weak | Moderate | Strong | Recall (%) |
|  | Inactive | 3688 (3416) | 950 (854) | 75 (73) | 171 (128) | 158 (113) | 73.1 (74.5) |
|  | Very Weak | 3 (3) | 37 (35) | 0 (0) | 3 (2) | 0 (0) | 86 (87.5) |
|  | Weak | 30 (23) | 83 (72) | 67 (67) | 27 (22) | 8 (5) | 31.2 (35.4) |
|  | Moderate | 0 (0) | 1 (1) | 0 (0) | 27 (24) | 6 (5) | 79.4 (80) |
|  | Strong | 0 (0) | 0 (0) | 0 (0) | 5 (5) | 26 (25) | 83.9 (83.3) |
|  | Precision (%) | 99.1 (99.2) | 3.5 (3.6) | 47.2 (47.9) | 11.6 (13.3) | 13.1 (16.9) |  |

Binding Accuracy: 70.7% or 72.2% when AD is applied.

**Table S9.** Confusion matrix for estrogen receptor binding activity from the values seen in the CERAPP evaluation set and the random forest model. Information for testing set substances with 4 or more sources. Numbers in brackets are when the AD is applied.

|  |  | Predicted | | | | |  |
| --- | --- | --- | --- | --- | --- | --- | --- |
| Observed |  | Inactive | Very Weak | Weak | Moderate | Strong | Recall (%) |
|  | Inactive | 939 (868) | 233 (207) | 16 (15) | 43 (35) | 43 (32) | 73.7 (75) |
|  | Very Weak | 1 (1) | 6 (6) | 0 (0) | 2 (1) | 0 (0) | 66.7 (75) |
|  | Weak | 6 (4) | 19 (17) | 11 (11) | 9 (9) | 3 (2) | 22.9 (25.6) |
|  | Moderate | 0 (0) | 0 (0) | 0 (0) | 3 (3) | 3 (3) | 50 (50) |
|  | Strong | 0 (0) | 0 (0) | 0 (0) | 1 (1) | 4 (4) | 80 (80) |
|  | Precision (%) | 99.3 (99.4) | 2.3 (2.6) | 47.9 (42.3) | 5.2 (6.1) | 7.5 (9.8) |  |

Binding Accuracy: 58.7% or 61.1% when AD is applied.

**Table S10.** Confusion matrix for estrogen receptor agonist activity from the values seen in the CERAPP evaluation set and the random forest model. Information for all substances. Numbers in brackets are when the AD is applied.

|  |  | Predicted | | | | |  |
| --- | --- | --- | --- | --- | --- | --- | --- |
| Observed |  | Inactive | Very Weak | Weak | Moderate | Strong | Recall (%) |
|  | Inactive | 5168 (4479) | 186 (173) | 355 (280) | 122 (96) | 61 (35) | 87.7 (88.5) |
|  | Very Weak | 2 (2) | 13 (12) | 4 (4) | 0 (0) | 0 (0) | 68.4 (66.7) |
|  | Weak | 36 (26) | 12 (8) | 109 (102) | 19 (19) | 3 (3) | 60.9 (64.6) |
|  | Moderate | 1 (1) | 0 (0) | 2 (2) | 28 (28) | 0 (0) | 90.3 (90.3) |
|  | Strong | 1 (0) | 0 (0) | 1 (1) | 3 (3) | 37 (36) | 88.1 (90) |
|  | Precision (%) | 99.2 (99.4) | 6.2 (6.2) | 23.1 (26.2) | 16.3 (19.2) | 36.6 (48.6) |  |

Binding Accuracy: 79.1% or 80.0% when AD is applied.

**Table S11.** Confusion matrix for estrogen receptor agonist activity from the values seen in the CERAPP evaluation set and the random forest model. Information for testing set substances. Numbers in brackets are when the AD is applied.

|  |  | Predicted | | | | |  |
| --- | --- | --- | --- | --- | --- | --- | --- |
| Observed |  | Inactive | Very Weak | Weak | Moderate | Strong | Recall (%) |
|  | Inactive | 1281 (1080) | 48 (46) | 87 (70) | 37 (30) | 12 (8) | 87.4 (87.5) |
|  | Very Weak | 2 (2) | 2 (2) | 4 (4) | 0 (0) | 0 (0) | 25 (25) |
|  | Weak | 11 (8) | 5 (3) | 27 (27) | 7 (7) | 1 (1) | 52.9 (58.7) |
|  | Moderate | 1 (1) | 0 (0) | 1 (1) | 4 (4) | 0 (0) | 66.7 (66.7) |
|  | Strong | 0 (0) | 0 (0) | 0 (0) | 0 (0) | 11 (10) | 100 (100) |
|  | Precision (%) | 98.9 (99) | 3.6 (3.9) | 26.2 (26.5) | 8.3 (9.8) | 45.8 (52.6) |  |

Binding Accuracy: 66.4% or 67.6% when AD is applied.

**Table S12.** Confusion matrix for estrogen receptor antagonist activity from the values seen in the CERAPP evaluation set and the random forest model. Information is for all substances. Numbers in brackets are when the AD is applied.

|  |  | Predicted | | | | |  |
| --- | --- | --- | --- | --- | --- | --- | --- |
| Observed |  | Inactive | Very Weak | Weak | Moderate | Strong | Recall (%) |
|  | Inactive | 4857 (4621) | 678 (631) | 596 (463) | 46 (31) | 44 (26) | 78.1 (80.1) |
|  | Very Weak | 4 (3) | 68 (63) | 3 (3) | 0 (0) | 1 (0) | 89.5 (91.3) |
|  | Weak | 27 (27) | 20 (19) | 129 (96) | 6 (5) | 6 (5) | 68.6 (63.2) |
|  | Moderate | 0 (0) | 0 (0) | 0 (0) | 10 (6) | 0 (0) | 100 (100) |
|  | Strong | 1 (1) | 0 (0) | 1 (0) | 0 (0) | 8 (6) | 80 (85.7) |
|  | Precision (%) | 99.3 (99.3) | 8.9 (8.8) | 17.7 (17.1) | 16.1 (14.3) | 13.6 (16.2) |  |

Binding Accuracy: 83.2% or 84.0% when AD is applied.

**Table S13.** Confusion matrix for estrogen receptor antagonist activity from the values seen in the CERAPP evaluation set and the random forest model. Information for testing set substances. Numbers in brackets are when the AD is applied.

|  |  | Predicted | | | | |  |
| --- | --- | --- | --- | --- | --- | --- | --- |
| Observed |  | Inactive | Very Weak | Weak | Moderate | Strong | Recall (%) |
|  | Inactive | 1194 (1114) | 184 (168) | 150 (107) | 12 (10) | 16 (13) | 76.7 (78.9) |
|  | Very Weak | 3 (3) | 16 (16) | 3 (3) | 0 (0) | 0 (0) | 72.7 (72.7) |
|  | Weak | 7 (7) | 6 (6) | 24 (19) | 3 (3) | 3 (2) | 55.8 (51.4) |
|  | Moderate | 0 (0) | 0 (0) | 0 (0) | 1 (0) | 0 (0) | 100.0 (NA) |
|  | Strong | 1 (1) | 0 (0) | 1 (0) | 0 (0) | 3 (3) | 60 (75) |
|  | Precision (%) | 99.1 (99) | 7.8 (8.4) | 17.1 (14.7) | 6.3 (0) | 13.6 (16.7) |  |

Binding Accuracy: 73.1% or 69.5% when AD is applied.

# Summary of Estrogen Receptor Activity Prediction

**Table S14.** Comparison of prediction statistic (balanced accuracies (%), coverage (%), recall (%), and precision (%)) for binary ER binding models. All values given are when ADs are applied.

| Model | Balanced Accuracy (%) | Coverage (%) | Recall (%) | Precision (%) |
| --- | --- | --- | --- | --- |
| RF Model (Evaluation Set) | 88.8 | 90.2 | 88.2 | 35.6 |
| RF Model (Test Set) | 88.1 | 88.5 | 87.6 | 38.2 |
| CERAPP Consensus Model | 79.9 | 100 | 67.1 | 38.9 |
| CaseUltra | 89.8 | 64.4 | 98.1 | 34.3 |
| ACD Percepta | 76.0 | 100 | 54.9 | 56.6 |
| ADMET Predictor | 72.6 | 90.6 | 56.8 | 25.2 |
| VEGA | 73.3 | 75.9 | 50.6 | 44.1 |

**Table S15.** Comparison of prediction statistic (balanced accuracies (%), coverage (%), recall (%), and precision (%)) for binary ER agonist models. All values given are when ADs are applied.

| Model | Balanced Accuracy (%) | Coverage (%) | Recall (%) | Precision (%) |
| --- | --- | --- | --- | --- |
| RF Model (Evaluation Set) | 86.4 | 89.7 | 86.6 | 28.0 |
| RF Model (Test Set) | 86.5 | 87.9 | 87.0 | 30.7 |
| CERAPP Consensus Model | 79.8 | 100 | 64.6 | 42.9 |
| CaseUltra ER Agonist | 90.1 | 69.4 | 97.6 | 31.9 |
| CaseUltra ER Agonist Alpha | 84.3 | 79.1 | 97.2 | 18.8 |
| CaseUltra ER Agonist Beta | 74.8 | 59.6 | 78.5 | 17.1 |

**Table S16.** Comparison of prediction statistic (balanced accuracies (%), coverage (%), recall (%), and precision (%)) for binary ER agonist models. All values given are when ADs are applied.

| Model | Balanced Accuracy (%) | Coverage (%) | Recall (%) | Precision (%) |
| --- | --- | --- | --- | --- |
| RF Model (Evaluation Set) | 85.4 | 88.9 | 89.6 | 15.2 |
| RF Model (Test Set) | 82.0 | 88.4 | 83.7 | 13.0 |
| CERAPP Consensus Model | 53.3 | 100 | 15.1 | 7.4 |
| CaseUltra ER Antagonist | 89.9 | 65.8 | 98.9 | 26.0 |
| CaseUltra ER Antagonist Alpha | 85.6 | 80.0 | 96.3 | 17.3 |
| CaseUltra ER Antagonist Beta | 78.1 | 69.6 | 92.1 | 12.0 |

**Table S17.** Comparison of prediction statistic (balanced accuracies (%) and coverage (%)) for multi-class ER binding models. All values given are when ADs are applied.

| Model | Balanced Accuracy (%) | Coverage (%) |
| --- | --- | --- |
| RF Model (Evaluation Set) | 72.2 | 90.8 |
| RF Model (Test Set) | 61.1 | 90.8 |
| CERAPP Consensus Model | 37.4 | 100 |
| UNISTRA InfoChim | 34.4 | 100 |
| LockheedMartin EPA 1 | 36.9 | 100 |
| LockheedMartin EPA 2 | 34.1 | 100 |

**Table S18.** Comparison of prediction statistic (balanced accuracies (%) and coverage (%)) for multi-class ER agonist models. All values given are when ADs are applied.

| Model | Balanced Accuracy (%) | Coverage (%) |
| --- | --- | --- |
| RF Model (Evaluation Set) | 80.0 | 86.2 |
| RF Model (Test Set) | 67.6 | 84.6 |
| CERAPP Consensus Model | 37.9 | 100 |
| UNISTRA InfoChim | 32.4 | 100 |
| LockheedMartin EPA 1 | 38.3 | 100 |
| LockheedMartin EPA 2 | 34.4 | 100 |

**Table S19.** Comparison of prediction statistic (balanced accuracies (%) and coverage (%)) for multi-class ER antagonist models. All values given are when ADs are applied.

| Model | Balanced Accuracy (%) | Coverage (%) |
| --- | --- | --- |
| RF Model (Evaluation Set) | 84.0 | 92.3 |
| RF Model (Test Set) | 69.5 | 90.7 |
| CERAPP Consensus Model | 21.2 | 100 |
| UNISTRA InfoChim | 22.5 | 100 |
| LockheedMartin EPA 1 | 21.9 | 100 |

# Confusion Matrices of Androgen Prediction

Below are the confusion matrices for the binary activity of the RFs models developed.

**Table S20.** Confusion matrix for androgen receptor binding activity from the values seen in the CoMPARA evaluation set and the random forest model. Information for all substances. Numbers in brackets are when the AD is applied.

|  |  | Predicted | | |  |
| --- | --- | --- | --- | --- | --- |
| Observed |  | Inactive | Active | Recall (%) | |
|  | Inactive | 2754 (2492) | 544 (415) | 83.5 (85.7) | |
|  | Active | 66 (56) | 374 (297) | 85.0 (84.1) | |
|  | Precision (%) | 97.7 (97.8) | 40.7 (41.1) |  | |

Binding Accuracy: 84.3% or 84.9% when AD is applied.

**Table S21.** Confusion matrix for estrogen receptor binding activity from the values seen in the CoMPARA evaluation set and the random forest model. Information for testing set. Numbers in brackets are when the AD is applied.

|  |  | Predicted | |  |
| --- | --- | --- | --- | --- |
| Observed |  | Inactive | Active | Recall (%) |
|  | Inactive | 677 (595) | 140 (107) | 82.9 (84.8) |
|  | Active | 19 (14) | 99 (79) | 83.9 (94.9) |
|  | Precision (%) | 97.3 (97.7) | 41.4 (42.5) |  |

Binding Accuracy: 83.4% or 84.9% when AD is applied.

**Table S22.** Confusion matrix for estrogen receptor agonist activity from the values seen in the CoMPARA evaluation set and the random forest model. Information for all substances. Numbers in brackets are when the AD is applied.

|  |  | Predicted | |  |
| --- | --- | --- | --- | --- |
| Observed |  | Inactive | Active | Recall (%) |
|  | Inactive | 4429 (3986) | 65 (46) | 98.6 (98.9) |
|  | Active | 20 (19) | 146 (132) | 88.0 (87.4) |
|  | Precision (%) | 99.6 (99.5) | 69.2 (74.2) |  |

Binding Accuracy: 93.3% or 93.1% when AD is applied

**Table S23.** Confusion matrix for estrogen receptor agonist activity from the values seen in the CoMPARA evaluation set and the random forest model. Information for testing set substances. Numbers in brackets are when the AD is applied.

|  |  | Predicted | |  |
| --- | --- | --- | --- | --- |
| Observed |  | Inactive | Active | Recall (%) |
|  | Inactive | 1108 (989) | 12 (9) | 98.9 (99.1) |
|  | Active | 6 (5) | 39 (37) | 86.7 (88.1) |
|  | Precision (%) | 99.5 (99.5) | 76.5 (80.4) |  |

Binding Accuracy: 92.8% or 93.6% when AD is applied

**Table S24.** Confusion matrix for estrogen receptor antagonist activity from the values seen in the CERAPP evaluation set and the random forest model. Information for all substances. Numbers in brackets are when the AD is applied.

|  |  | Predicted | |  |
| --- | --- | --- | --- | --- |
| Observed |  | Inactive | Active | Recall |
|  | Inactive | 3005 (2758) | 534 (416) | 84.9 (86.9) |
|  | Active | 31 (27) | 312 (236) | 91.0 (89.7) |
|  | Precision (%) | 99.0 (99.0) | 36.9 (36.2) |  |

Binding Accuracy: 87.9% or 88.3% when AD is applied

**Table S25.** Confusion matrix for estrogen receptor antagonist activity from the values seen in the CERAPP evaluation set and the random forest model. Information for testing set substances. Numbers in brackets are when the AD is applied.

|  |  | Predicted | |  |
| --- | --- | --- | --- | --- |
| Observed |  | Inactive | Active | Recall (%) |
|  | Inactive | 732 (669) | 159 (118) | 82.2 (85.0) |
|  | Active | 18 (15) | 62 (4) | 77.5 (78.9) |
|  | Precision (%) | 97.6 (97.8) | 28.1 (25.3) |  |

Binding Accuracy: 79.8% or 78.9% when AD is applied

**Table S26.** Confusion matrix for androgen receptor binding activity from the values seen in the CoMPARA evaluation set and the random forest model. Information for all substances. Numbers in brackets are when the AD is applied.

|  |  | Predicted | | | | |  |
| --- | --- | --- | --- | --- | --- | --- | --- |
| Observed |  | Inactive | Very Weak | Weak | Moderate | Strong | Recall (%) |
|  | Inactive | 3688 (3416) | 950 (854) | 75 (73) | 171 (128) | 158 (113) | 73.1 (74.5) |
|  | Very Weak | 3 (3) | 37 (35) | 0 (0) | 3 (2) | 0 (0) | 86 (87.5) |
|  | Weak | 30 (23) | 83 (72) | 67 (67) | 27 (22) | 8 (5) | 31.2 (35.4) |
|  | Moderate | 0 (0) | 1 (1) | 0 (0) | 27 (24) | 6 (5) | 79.4 (80) |
|  | Strong | 0 (0) | 0 (0) | 0 (0) | 5 (5) | 26 (25) | 83.9 (83.3) |
|  | Precision (%) | 99.1 (99.2) | 3.5 (3.6) | 47.2 (47.9) | 11.6 (13.3) | 13.1 (16.9) |  |

Binding Accuracy: 75.8% or 75.5% when AD is applied.

**Table S27.** Confusion matrix for androgen receptor binding activity from the values seen in the CoMPARA evaluation set and the random forest model. Information for testing set substances. Numbers in brackets are when the AD is applied.

|  |  | Predicted | | | | |  |
| --- | --- | --- | --- | --- | --- | --- | --- |
| Observed |  | Inactive | Very Weak | Weak | Moderate | Strong | Recall (%) |
|  | Inactive | 939 (868) | 233 (207) | 16 (15) | 43 (35) | 43 (32) | 73.7 (75) |
|  | Very Weak | 1 (1) | 6 (6) | 0 (0) | 2 (1) | 0 (0) | 66.7 (75) |
|  | Weak | 6 (4) | 19 (17) | 11 (11) | 9 (9) | 3 (2) | 22.9 (25.6) |
|  | Moderate | 0 (0) | 0 (0) | 0 (0) | 3 (3) | 3 (3) | 50 (50) |
|  | Strong | 0 (0) | 0 (0) | 0 (0) | 1 (1) | 4 (4) | 80 (80) |
|  | Precision (%) | 99.3 (99.4) | 2.3 (2.6) | 47.9 (42.3) | 5.2 (6.1) | 7.5 (9.8) |  |

Binding Accuracy: 60.0% or 57.5% when AD is applied.

**Table S28.** Confusion matrix for androgen receptor agonist activity from the values seen in the CoMPARA evaluation set and the random forest model. Information for all substances. Numbers in brackets are when the AD is applied.

|  |  | Predicted | | | | | |  | |
| --- | --- | --- | --- | --- | --- | --- | --- | --- | --- |
| Observed |  | Inactive | Very Weak | Weak | Moderate | Strong | Recall (%) | |  |
|  | Inactive | 2302 (2141) | 673 (611) | 280 (207) | 2 (2) | 39 (22) | 69.8 (71.8) | |  |
|  | Very Weak | 16 (15) | 122 (112) | 1 (0) | 0 (0) | 2 (2) | 86.5 (86.8) | |  |
|  | Weak | 21 (16) | 29 (26) | 113 (87) | 14 (12) | 39 (33) | 52.3 (50) | |  |
|  | Moderate | 0 (0) | 0 (0) | 2 (1) | 11 (8) | 2 (2) | 73.3 (72.7) | |  |
|  | Strong | 0 (0) | 0 (0) | 1 (1) | 1 (1) | 61 (52) | 96.8 (96.3) | |  |
|  | Precision (%) | 98.4 (98.6) | 14.8 (15) | 28.5 (29.4) | 39.3 (34.8) | 42.7 (46.8) |  | |  |

Binding Accuracy: 71.2% or 71.6% when AD is applied.

**Table S29.** Confusion matrix for androgen receptor agonist activity from the values seen in the CoMPARA evaluation set and the random forest model. Information for testing set substances. Numbers in brackets are when the AD is applied.

|  |  | Predicted | | | | |  |
| --- | --- | --- | --- | --- | --- | --- | --- |
| Observed |  | Inactive | Very Weak | Weak | Moderate | Strong | Recall (%) |
|  | Inactive | 564 (518) | 173 (168) | 71 (107) | 0 (10) | 9 (13) | 69 (63.5) |
|  | Very Weak | 7 (3) | 25 (16) | 1 (3) | 0 (0) | 1 (0) | 73.5 (72.7) |
|  | Weak | 9 (7) | 11 (6) | 22 (19) | 5 (3) | 13 (2) | 36.7 (51.4) |
|  | Moderate | 0 (0) | 0 (0) | 2 (0) | 2 (0) | 2 (0) | 33.3 (NA) |
|  | Strong | 0 (1) | 0 (0) | 1 (0) | 1 (0) | 14 (3) | 87.5 (75) |
|  | Precision (%) | 97.2 (97.9) | 12 (8.4) | 29.4 (14.7) | 25 (0) | 35.9 (16.7) |  |

Binding Accuracy: 70.1% or 71.2% when AD is applied.

**Table S30.** Confusion matrix for androgen receptor antagonist activity from the values seen in the CoMPARA evaluation set and the random forest model. Information for all substances. Numbers in brackets are when the AD is applied.

|  |  | Predicted | | | | |  |
| --- | --- | --- | --- | --- | --- | --- | --- |
| Observed |  | Inactive | Very Weak | Weak | Moderate | Strong | Recall (%) |
|  | Inactive | 2793 (2611) | 608 (537) | 36 (19) | 33 (24) | 69 (24) | 78.9 (81.2) |
|  | Very Weak | 21 (18) | 121 (111) | 2 (2) | 0 (0) | 4 (2) | 81.8 (83.5) |
|  | Weak | 30 (26) | 48 (41) | 76 (50) | 5 (1) | 17 (9) | 43.2 (39.4) |
|  | Moderate | 0 (0) | 1 (1) | 0 (0) | 5 (3) | 0 (0) | 83.3 (75) |
|  | Strong | 0 (0) | 0 (0) | 0 (0) | 0 (0) | 10 (5) | 100 (100) |
|  | Precision (%) | 98.2 (98.3) | 15.6 (16.1) | 66.7 (70.4) | 11.6 (10.7) | 10 (12.5) |  |

Binding Accuracy: 77.4% or 75.8% when AD is applied

**Table S31.** Confusion matrix for androgen receptor antagonist activity from the values seen in the CoMPARA evaluation set and the random forest model. Information for testing set substances. Numbers in brackets are when the AD is applied.

|  |  | Predicted | | | | | |  | |
| --- | --- | --- | --- | --- | --- | --- | --- | --- | --- |
| Observed |  | Inactive | Very Weak | Weak | Moderate | Strong | Recall (%) | |  |
|  | Inactive | 697 (649) | 147 (127) | 7 (2) | 11 (7) | 17 (5) | 79.3 (82.2) | |  |
|  | Very Weak | 7 (6) | 32 (30) | 2 (2) | 0 (0) | 0 (0) | 78 (78.9) | |  |
|  | Weak | 6 (4) | 19 (13) | 11 (10) | 4 (1) | 7 (4) | 23.4 (31.3) | |  |
|  | Moderate | 0 (0) | 1 (1) | 0 (0) | 0 (0) | 0 (0) | 0 (0) | |  |
|  | Strong | 0 (0) | 0 (0) | 0 (0) | 0 (0) | 2 (0) | 100 (NA) | |  |
|  | Precision (%) | 98.2 (98.5) | 16.1 (17.5) | 70.4 (71.4) | 0 (0) | 7.7 (0) |  | |  |

Binding Accuracy: 56.1% or 48.1% when AD is applied

# Summary of Androgen Receptor Activity Prediction

**Table S32.** Comparison of prediction statistic (balanced accuracies (%), coverage (%), recall (%), and precision (%)) for binary AR binding models. All values given are when ADs are applied.

| Model | Balanced Accuracy (%) | Coverage (%) | Recall (%) | Precision (%) |
| --- | --- | --- | --- | --- |
| RF Model (Evaluation Set) | 87.2 | 84.9 | 84.1 | 41.7 |
| RF Model (Test Set) | 85.0 | 84.9 | 84.9 | 42.4 |
| CoMPARA Consensus Model | 100.0 | 76.9 | 64.3 | 45.1 |
| ADMET | 84.1 | 71.1 | 59.1 | 28.0 |
| VEGA | 85.0 | 66.5 | 35.9 | 58.2 |
| Oasis TIMES | 93.0 | 62.1 | 27.9 | 45.8 |

**Table S33.** Comparison of prediction statistic (balanced accuracies (%), coverage (%), recall (%), and precision (%)) for binary AR agonist models. All values given are when ADs are applied.

| Model | Balanced Accuracy (%) | Coverage (%) | Recall (%) | Precision (%) |
| --- | --- | --- | --- | --- |
| RF Model (Evaluation Set) | 93.1 | 89.8 | 87.4 | 74.2 |
| RF Model (Test Set) | 93.6 | 89.3 | 88.1 | 80.4 |
| CoMPARA Consensus Model | 84.7 | 100.0 | 72.3 | 48.6 |
| Oasis TIMES | 72.8 | 71.8 | 49.6 | 30.9 |
| CaseUltra Agonist HEK | 80.6 | 43.7 | 94.5 | 17.9 |
| CaseUltra Agonist MDA | 93.6 | 67.6 | 98.7 | 30.9 |

**Table S34.** Comparison of prediction statistic (balanced accuracies (%), coverage (%), recall (%), and precision (%)) for binary AR antagonist models. All values given are when ADs are applied.

| Model | Balanced Accuracy (%) | Coverage (%) | Recall (%) | Precision (%) |
| --- | --- | --- | --- | --- |
| RF Model (Evaluation Set) | 88.3 | 88.5 | 89.7 | 36.2 |
| RF Model (Test Set) | 78.9 | 86.7 | 72.7 | 25.3 |
| CoMPARA Consensus Model | 73.3 | 100.0 | 59.8 | 30.6 |
| Oasis TIMES | 58.8 | 87.8 | 21.4 | 31.6 |
| CaseUltra Agonist HEK | 87.7 | 75.6 | 91.7 | 39.3 |
| CaseUltra Agonist MDA | 83.4 | 75.3 | 92.8 | 29.3 |

**Table S35.** Comparison of prediction statistic (balanced accuracies (%) and coverage (%)) for multi-class AR binding models. All values given are when ADs are applied.

| Model | Balanced Accuracy (%) | Coverage (%) |
| --- | --- | --- |
| RF Model (Evaluation Set) | 75.5 | 89.8 |
| RF Model (Test Set) | 57.5 | 88.1 |
| TUM | 32.1 | 99.9 |
| Jussieu | 30.5 | 99.9 |
| VCCLAB | 35.5 | 97.0 |
| UNISTRA | 37.6 | 82.2 |
| LM | 18.4 | 8.9 |

**Table S36.** Comparison of prediction statistic (balanced accuracies (%) and coverage (%)) for multi-class AR agonist models. All values given are when ADs are applied.

| Model | Balanced Accuracy (%) | Coverage (%) |
| --- | --- | --- |
| RF Model (Evaluation Set) | 71.6 | 89.5 |
| RF Model (Test Set) | 71.2 | 87.7 |
| TUM | 28.4 | 99.9 |
| Jussieu | 26.0 | 99.9 |
| VCCLAB | 33.2 | 96.9 |
| UNISTRA | 37.0 | 82.2 |
| LM | 18.8 | 8.9 |

**Table S37.** Comparison of prediction statistic (balanced accuracies (%) and coverage (%)) for multi-class AR antagonist models. All values given are when ADs are applied.

| Model | Balanced Accuracy (%) | Coverage (%) |
| --- | --- | --- |
| RF Model (Evaluation Set) | 75.8 | 89.8 |
| RF Model (Test Set) | 48.1 | 88.8 |
| TUM | 17.8 | 9.0 |
| VCCLAB | 21.5 | 99.9 |
| LM | 25.4 | 97.3 |

# References

(1) United States Enviromental Protection Agency. *CompTox Chemicals Dashboard*; 2017.

(2) Kim, S.; Thiessen, P. A.; Bolton, E. E.; et al. PubChem Substance and Compound Databases. *Nucleic Acids Res.* **2016**, *44* (D1), D1202–D1213. https://doi.org/10.1093/nar/gkv951.

(3) Pence, H. E.; Williams, A. ChemSpider: An Online Chemical Information Resource. *J. Chem. Educ.* **2010**, *87* (11), 1123–1124. https://doi.org/10.1021/ed100697w.

(4) Judson, R. S.; Martin, M. T.; Egeghy, P.; et al. Aggregating Data for Computational Toxicology Applications: The U.S. Environmental Protection Agency (EPA) Aggregated Computational Toxicology Resource (ACToR) System. *Int. J. Mol. Sci.* **2012**, *13* (2), 1805–1831. https://doi.org/10.3390/ijms13021805.

(5) Mansouri, K.; Abdelaziz, A.; Rybacka, A.; et al. CERAPP: Collaborative Estrogen Receptor Activity Prediction Project. *Environ. Health Perspect.* **2016**, *124* (7), 1023–1033. https://doi.org/10.1289/ehp.1510267.

(6) Mansouri, K.; Kleinstreuer, N.; Abdelaziz, A. M.; et al. CoMPARA: Collaborative Modeling Project for Androgen Receptor Activity. *Environ. Health Perspect.* **2020**, *128* (2), 027002. https://doi.org/10.1289/EHP5580.
